# State-Level Geographic Disparities in Liver Transplant Access: Waitlist Outcome Patterns

**DOI:** 10.3390/jcm15114212

**Published:** 2026-05-29

**Authors:** Ahmed Nahian, Lisa McFadden, Tanzina Ela

**Affiliations:** 1 Lake Erie College of Osteopathic Medicine at Seton Hill, Greensburg, PA 15601, USA; ahmed.nahian.usa@gmail.com; 2 Sanford School of Medicine, University of South Dakota, Vermillion, SD 57069, USA; 3 Department of Internal Medicine, South Brooklyn Health, New York, NY 11235, USA

**Keywords:** liver transplantation, geographic disparities, rural health, waitlist mortality, OPTN, UNOS, ecological study

## Abstract

**Background/Objectives**: Geographic inequity remains a persistent concern in liver transplantation, particularly for patients requiring liver transplantation for advanced chronic liver disease, in which transplantation remains the definitive therapy for advanced disease. We evaluated state-level differences in liver transplant waitlist burden using publicly available U.S. data. **Methods**: We performed a retrospective ecological panel study using publicly available United Network for Organ Sharing (UNOS)-derived annual state tables from 1995 to 2025. Six analyzable states were grouped as higher-rurality/substantial-rural-population states (Texas, North Carolina, Pennsylvania) and urban-dominant states (California, New Jersey, Massachusetts). Primary outcomes were annual liver transplants, death removals, and death-share (death removals divided by death removals plus transplants). Descriptive comparisons, era analyses, and heteroscedasticity-robust regression models were performed. **Results**: The final dataset contained 186 state-year observations. Across 1995–2025, higher-rurality states had more cumulative transplants than urban-dominant states (39,471 vs. 34,178) and fewer cumulative death removals (8642 vs. 10,625). Mean death-share was lower in higher-rurality states (18.7% vs. 22.6%), as was the death-to-transplant ratio (0.219 vs. 0.311). From 2020 to 2025, higher-rurality states again demonstrated lower mean death-share (9.5% vs. 14.3%). In regression modeling, higher-rurality group membership was associated with lower death-share (β = −0.0389, 95% CI −0.0604 to −0.0175, *p* < 0.001), while the post-2020 era was independently associated with lower death-share (β = −0.1091, 95% CI −0.1299 to −0.0882, *p* < 0.001). Highly rural low-volume states initially considered for analysis had sparse or suppressed counts and could not be reliably modeled. **Conclusions**: In this six-state ecological study, higher-rurality states with substantial rural populations exhibited lower waitlist death-removal burden than urban-dominant comparators. These discoveries probably indicate the varying transplant-system configurations instead of the individual rural access being better. The ecological data related to the public can be the basis for significant hypotheses concerning the transplant discrepancies, but the exhaustive consecutive tasks need to be supplemented by static national studies that are patient-level and relevant to rurality, travel distance, PSC-specific cohorts, and psychosocial determinants.

## 1. Introduction

Liver transplantation in the United States is undergoing a rapid transformation in the medical field, while also changing the epidemiologic and policy environment that led to changes in both the number of people needing organs and the expectations of equal access. The first national data registry shows that transplant activity has been at an all-time high due to the improvement in various factors, including techniques, perioperative management, donor utilization, and multidisciplinary candidate selection for surgery [1,2,3,4]. Concurrently, the problem of chronic liver disease is still persistent, with factors such as cirrhosis, alcohol-related liver disease, metabolic dysfunction-associated steatotic liver disease, viral hepatitis, and sequelae, such as autoimmune cholestatic disorder, and liver biliary cancer [1,5,6]. Liver transplantation remains the only definitive therapy for end-stage liver disease; the pathway from referral to transplantation functions as a critical national system for preventing mortality [2,3]. Each stage in this pathway contributes meaningfully to preventing mortality, reducing decompensation, and limiting recurrent hospitalization. On the other hand, federal oversight bodies and transplant stakeholders, seeing the problems, have increasingly spoken up about nonmedical factors, for instance, those concerning region, distance, local supply–demand mismatch, and center-specific access patterns, that need to be tackled so that all patients are treated equally [2,3,4]. The focus of this policy unveils a more subtle message: surgical efficacy alone would not suffice, and patients must also be able to reach the transplant system on time. In this sense, analyses that discuss how geography deals with transplant access will always remain of great interest to doctors, administrators, and public health specialists alike.

The liver transplantation geographic difference used to be primarily dictated by local allocation boundaries, the amount of organ provided, and the unequal distribution of transplant centers. Before the changes in laws, studies found stark contrasts suffered by patients of median End-Stage Liver Disease (MELD) scores at transplantation, waitlist mortality, and organ availability in different regions based on donor service areas, and fixed regional structures [2,7,8]. The Organ Procurement and Transplantation Network (OPTN) has made efforts to improve equity in organ allocation, particularly through policy changes aimed at reducing geographic disparities in access to transplantation. It punctually applied the Acuity Circles policy in February 2020, which will then this year replace the previous boundary, which is a distance-based framework with priority for medical urgency, thus reducing from the arbitrary geographic disparities postulated [3,9]. The registry analyses that came after were noted to have shown specific changes in the distribution of organs and the addition of donors as well as the returning of waitlisters to baseline levels, but the longer-term effects are still underway [9,10,11]. In addition, the transplant arena follows closely the economic model, which leads to the constant distribution being embraced, a model that includes various candidate characteristics rather than being dependent solely on rigid historical geography [2,4]. It is, therefore, worthwhile to focus our attentions on the current period, where the state patterns observed after 2020 will likely exhibit dissimilarities from the previous decades. For this, a geographic analysis that spans both the old and the new policy eras can provide vital insights into whether public growth trajectories align with the roadmap of expected reforms. Such analyses, while unable to ascertain causation on their own, can still uncover signals that merit further examination in the patient realm.

Rural residency is among the topics most neglected and unexplored as prerequisites to transplant access. Transplant patients residing in less populated areas face the challenges of longer distances to travel, having fewer specialized physicians, limited referral pathways, poor transportation, and basic workforce shortages, along with reduced access to these facilities, becoming the hindrances to achieving their goals [12,13,14,15]. Literature from the transplant field in the past has shown that patients from rural areas would have a lower chance of being placed on a waitlist or receiving a transplant when compared to their counterparts in urban settings, whereas more recent research has centered on the issue that the gap might be the result of the structural elements rather than geography by itself such as the poverty index, proximity of the center, broadband limitation, and referral inefficiencies [13,14,15,16,17]. Aside from that, rurality is not binary. A state may have a relatively small proportion of rural residents while still containing a large absolute number of rural individuals due to overall population size, whereas on the contrary, a state may be very rural by proportion, but there is too little transplant volume for proper public reporting. This has some practical elements in terms of the use of publicly available statistics. It states that the analyses, which solely depend on percentage-rural rankings, may overlook places where large concentrations of rural patients actually live and seek care. Accordingly, during the potential ecological approach, the choice of which to adopt might come out of a mixture of the rural composition and of the transplant-system scale.

Primary sclerosing cholangitis (PSC) represents one example of a chronic liver disease in which transplantation remains an important therapeutic consideration. PSC is a chronic cholestatic disorder that is characterized by the recurrent loss of intrahepatic and/or extrahepatic bile duct and vasculature, causing biliary obstructive jaundice, recurrent cholangitis, secondary biliary cirrhosis, portal vein thrombosis, liver decompensation, and cholangiocarcinoma [18,19,20,21]. The disease is common in both adolescents and middle-aged adults and has been associated closely with inflammatory bowel disease, most commonly ulcerative colitis, which is linked to the long-term medical complexity of life in general [18,19]. No medical treatment has been found that clearly changes the natural history of advanced PS; therefore, immunologic and antifibrotic therapies are being researched. Liver transplantation is still the only treatment for end-stage PSC or PSC recurrence due to cholangitis, decompensation, or some malignancies [19,20,21,22]. While recurrence of PSC and graft-related complications can occur after transplantation, overall post-transplant outcomes remain favorable in most patients [20,21,22]. Due to their relatively young age and their high degree of medical engagement before they even reached transplant thresholds, many patients with PSC are affected by delays that may be associated with structural inefficiencies. Consequently, the uneven functioning of a system, which may be influenced by geographical factors, may lead to more than just generic waitlist statistics. However, the present analyses were not PSC-specific and therefore should not be interpreted as direct evidence regarding PSC-specific transplant outcomes or allocation equity.

The datasets that are published for transplant become an important opportunity for an exploration of these issues from a health systems point of view. The optimal state-year data will still be the ones that will cover patient-level registries since they are better suited for causal inference and risk adjustment, but the results based on a state-year level will still highlight the fact that organ transplant ecosystems in the US states are different, especially when they are analyzed over a period of decades. State-year data provide a framework for hypothesis generation regarding temporal state-level differences in transplant outcomes that may be influenced by rural–urban population composition. These data sources also provide the platform for replication through publicly available datasets generated from UNOS, together with the utilization of other sources other than institutional datasets of proprietary nature. The present study focused on the geographic distribution of transplant activity in the context of rural counties and cities. The geographic focus was on the United States, with particular attention to states with substantial rural populations (Texas, North Carolina, and Pennsylvania) compared with urban-dominant states (California, New Jersey, and Massachusetts), analyzed over the period 1995–2025. The main aim was to evaluate different burdens of death-share and the count of death-removing surgeries, which pragmatically reveals the number of death removals relative to the total number of deaths, including transplants performed. The secondary aim was to learn whether the post-2020 area was perceived to be distinct from previous times due to the change in national allocation conditions. As a hypothesis, the geography at the state level would offer a strong link to the differences in waitlist outcomes, even if we note that ecological interactions and patient experiences may diverge from each other and thus should be read accordingly.

## 2. Materials and Methods

### 2.1. Study Design and Data Source

Using Python, we performed a retrospective ecological panel study using the provided data of publicly available United Network for Organ Sharing (UNOS)-derived annual state-level tables. The Excel workbooks from the federally contracted nonprofit organization contained annual state counts for liver transplants and death removals by year. For liver transplants, the analytic row used was the workbook’s top-line “All Recipient Ages/All ABO” total for each state-year. For death removals, the corresponding analytic row was the workbook’s top-line “All Diagnosis/All Ages/All BMI” total, representing aggregate waitlist death removals across all diagnostic categories. We restricted the analytic period to 1995–2025 because death-removal tables began in 1995 and 2026 counts were explicitly “to date” and therefore incomplete.

### 2.2. State Selection and Grouping Rationale

**Figure 1:**
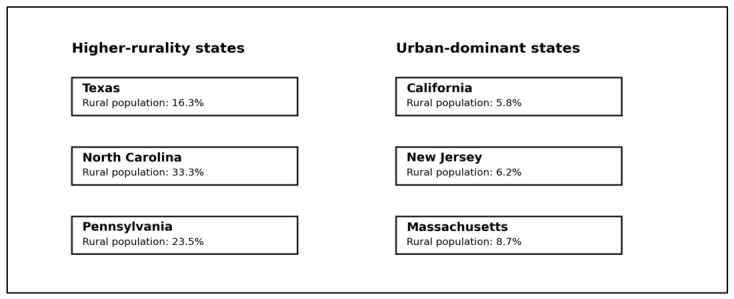
Study design schematic and state grouping. Schematic showing the six included states, classified as higher-rurality/substantial-rural-population (Texas, North Carolina, Pennsylvania) or urban-dominant (California, New Jersey, Massachusetts).

The initial project curation strategy preclassified states into three geographic archetypes: rural-dominant, mixed/high-volume, and urban-dominant. However, the proposed rural-dominant states (Vermont, Maine, and West Virginia) had nonreportable counts in the public-source extraction framework and were therefore not used for inferential comparisons. The final analyzable set comprised six states divided into two groups: higher-rurality/substantial-rural-population states (Texas, North Carolina, Pennsylvania) and urban-dominant states (California, New Jersey, Massachusetts). Rural-population percentages used for grouping were derived from U.S. Census Bureau urban–rural classifications, in which “rural” is defined as all population, housing, and territory not included within urban areas (defined as densely settled cores containing at least 2000 housing units or a population of at least 5000) [23]. As Figure 1 shows, state-level rural-population percentages were obtained from Census-derived summary data and used as a fixed characteristic for grouping: Texas 16.3%, North Carolina 33.3%, Pennsylvania 23.5%, California 5.8%, New Jersey 6.2%, and Massachusetts 8.7%.

This grouping emphasizes substantial rural population volume rather than percentage-only rurality and is consistent with the larger national observation that a state can be highly urbanized by percentage while still containing a large absolute rural population [6,23,24].

### 2.3. Variable Definitions

The primary raw outcomes were annual liver transplants and annual death removals. We defined death-share as:

$${\mathrm{death}\text{-}\mathrm{share}}_{s,t}=\frac{{\mathrm{death}\;\mathrm{removals}}_{s,t}}{{\mathrm{death}\;\mathrm{removals}}_{s,t}+{\mathrm{transplants}}_{s,t}}$$

where *s* denotes state and *t* denotes year. This measure was selected to normalize death-removal burden to the volume of observed definitive waitlist exits represented in the state tables. We also calculated the death-to-transplant ratio as cumulative death removals divided by cumulative transplants for descriptive group comparisons.

### 2.4. Handling of Suppressed/Null Data

No numerical imputation was performed. The six included states had complete analyzable values from 1995 through 2025 for the selected top-line transplant and death-removal rows. By contrast, the originally planned rural-dominant comparator states were excluded from inferential analyses because the project’s source materials indicated nonreportable or suppressed counts that would have yielded unstable estimates. This exclusion is a limitation but also an important systems-level observation: the most rural, low-population states may be underrepresented in public state-year transplant reporting.

### 2.5. Statistical Analysis

We summarized state-level totals and means over the full 1995–2025 period and over a contemporary post-2020 period. We also described four eras: 1995–2004, 2005–2014, 2015–2019, and 2020–2025. For inferential ecological modeling, we fit heteroscedasticity-robust ordinary least squares models with:

- death-share ~ higher-rurality group + post-2020 indicator;
- death-share ~ state rural-percentage + post-2020 indicator;
- annual transplants ~ higher-rurality group + post-2020 indicator.

The post-2020 indicator equaled 1 for years 2020–2025 and 0 otherwise. Robust HC1 standard errors were used. Because rural-percentage was operationalized as a fixed state-level characteristic using summary Census-derived estimates, models using continuous rurality did not include state fixed effects; instead, state-level ecological exposure was modeled directly. All tests were two-sided with α = 0.05. The analytic period was restricted to complete calendar-year tables available in the public UNOS/OPTN state-level data source at the time of extraction. Data for 2025 were included only if reported as complete annual counts in the downloaded state tables; partial or “to date” 2026 values were excluded. Because the study used aggregate public state-year tables rather than patient-level registry files, individual listing dates, event times, MELD/PELD scores, diagnosis-specific transplant indications, insurance status, race/ethnicity, comorbidities, transplant-center identifiers, ZIP codes, and Donation Service Area or Acuity Circle-level allocation units were not available for modeling. The analysis was therefore designed as an exploratory ecological analysis of state-level outcome patterns rather than a patient-level time-to-event analysis. Cox proportional hazards models, Fine-Gray competing-risk models, and multivariable patient-level adjustment were not feasible using the available aggregate public tables because the necessary individual-level covariates, event times, and competing-event indicators were not included.

### 2.6. Software and Reproducibility

Analyses were performed in Python 3.13.5 with pandas 2.2.3, NumPy 2.3.5, openpyxl 3.1.5, statsmodels 0.14.6, and matplotlib 3.10.8.

## 3. Results

### 3.1. Cohort Construction and Overall Panel

The final dataset contained 186 state-year observations: 6 analyzable states observed across 31 complete calendar years (1995–2025). Table 1 summarizes the study panel.

Across the full period, the three higher-rurality states contributed 39,471 cumulative transplants and 8642 cumulative death removals, whereas the three urban-dominant states contributed 34,178 cumulative transplants and 10,625 cumulative death removals. At the state level, cumulative transplants ranged from 1982 in New Jersey to 25,267 in California, while cumulative death removals ranged from 376 in New Jersey to 7929 in California. Mean death-share ranged from 16.0% in New Jersey to 27.5% in Massachusetts; among the higher-rurality states, mean death-share ranged from 18.0% in North Carolina to 19.1% in Pennsylvania. All study-specific values in this section were recalculated directly from the provided workbook. Because these metrics are not normalized per capita, comparisons may reflect differences in underlying population size in addition to transplant-system characteristics.

### 3.2. Contemporary Post-2020 Patterns

Table 2 shows post-2020 results.

From 2020 through 2025, California had the largest cumulative transplant volume (6847) of the six states, followed by Texas (5889) and Pennsylvania (3582). Over the same period, cumulative death removals were 976 in California, 768 in Texas, and 384 in Pennsylvania. Mean post-2020 death-share was lowest in North Carolina (7.0%), followed by Pennsylvania (9.7%), Texas (11.7%), California (12.6%), Massachusetts (14.9%), and New Jersey (15.3%). At the group level, the higher-rurality states had a lower post-2020 mean death-share than the urban-dominant states (9.5% vs. 14.3%) and a lower death-to-transplant ratio (0.114 vs. 0.148).

### 3.3. Group-Level Comparison

Over the entire 1995–2025 panel, the higher-rurality group had 15.5% more cumulative transplants than the urban-dominant group (39,471 vs. 34,178) and 18.7% fewer cumulative death removals (8642 vs. 10,625). Mean annual transplant counts were 424.4 in the higher-rurality group and 367.5 in the urban-dominant group, while mean annual death removals were 92.9 and 114.2, respectively. Mean death-share differed by −3.9 percentage points (18.7% vs. 22.6%), and the death-to-transplant ratio differed by −0.092 (0.219 vs. 0.311) (Table 3).

### 3.4. Era Trends and Visual Patterning

**Figure 2:**
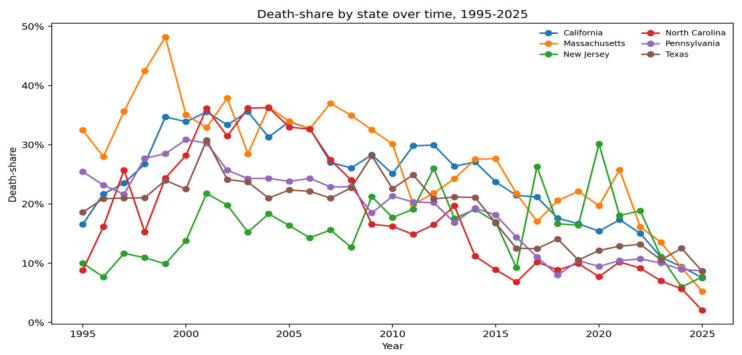
Annual death-share by state, 1995–2025. Annual death-share, defined as death removals ÷ (death removals + transplants), for each included state from 1995 through 2025.

Era summaries showed a monotonic reduction in group mean death-share over time, especially after 2015. In the higher-rurality group, mean death-share declined from 24.9% (1995–2004) to 21.7% (2005–2014), 11.5% (2015–2019), and 9.5% (2020–2025). In the urban-dominant group, the corresponding values were 26.3%, 25.4%, 19.7%, and 14.3%. Figure 2 shows that all six states experienced marked declines in death-share from 2020 to 2025, with the largest absolute drops in New Jersey (−22.5 percentage points) and Massachusetts (−14.5 percentage points).

**Figure 3:**
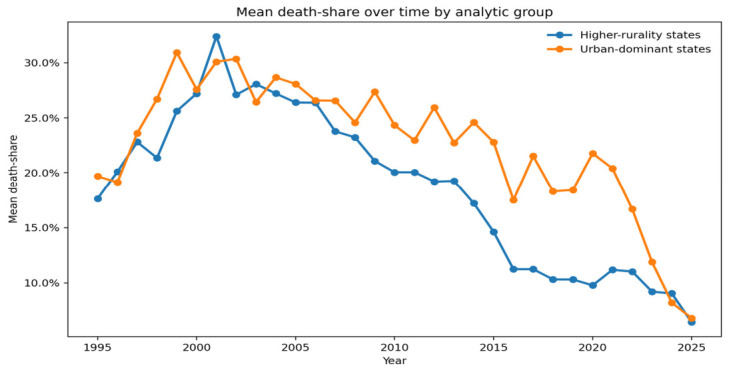
Group mean death-share over time. Comparison of the annual mean death-share of the higher-rurality and urban-dominant state groups.

Figure 3 shows that the urban-dominant group began 2020 with a substantially higher mean death-share than the higher-rurality group (21.8% vs. 9.8%) but that both groups converged to similarly low values by 2025 (6.8% vs. 6.5%).

**Figure 4:**
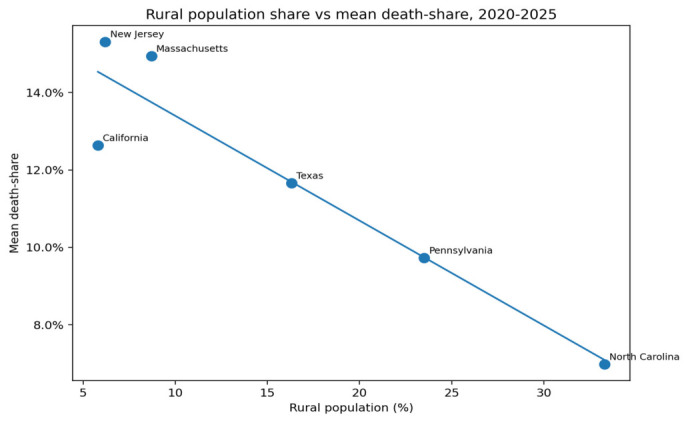
Rurality versus mean post2020 death-share. Scatterplot of state rural-population percentage against mean death-share for years 2020–2025.

Figure 4 shows an inverse cross-state association between rural-population percentage and mean post-2020 death-share in this selected sample.

**Figure 5:**
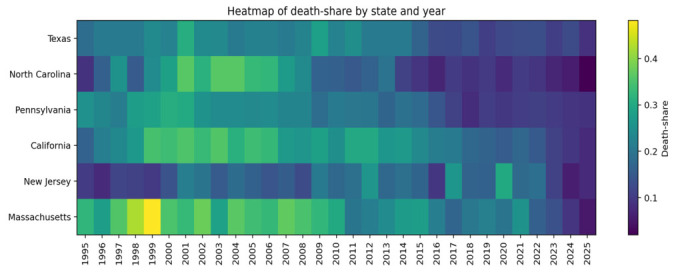
Heatmap of annual death-share by state and year. Heatmap with states on the *y*-axis, study year on the *x*-axis, and cell color representing annual death-share. This figure visually summarizes temporal convergence toward lower death-share in the post-2020 period.

Figure 5 displays the same pattern longitudinally as a state-by-year heatmap.

### 3.5. Regression Modeling

Table 4 presents ecological regression results.

In Model 1, higher-rurality group membership was associated with a lower death-share (β = −0.0389, 95% CI −0.0604 to −0.0175, *p* < 0.001), while the post-2020 era was independently associated with lower death-share (β = −0.1091, 95% CI −0.1299 to −0.0882, *p* < 0.001; R^2^ = 0.290). In Model 2, each 1-point higher state rural-population percentage was associated with a 0.001649 lower death-share (95% CI −0.002837 to −0.000461, *p* = 0.007), equivalent to an approximately 1.65-percentage-point lower death-share per 10-point increase in rurality, again with an independent post-2020 decline (β = −0.1091, 95% CI −0.1298 to −0.0883, *p* < 0.001; R^2^ = 0.276). In Model 3, post-2020 was associated with higher annual transplant counts (β = 220.8, 95% CI 86.9–354.8, *p* = 0.001); however, these findings are based on unadjusted state-level counts and may reflect differences in underlying population size rather than transplant-system performance alone. Higher-rurality group membership was not significantly associated with annual transplant volume (β = 56.9, 95% CI −25.0 to 138.8, *p* = 0.173; R^2^ = 0.095).

## 4. Discussion

This six-state ecological analysis offers a deliberately cautious but important interpretation of geographic variation in liver transplantation. The central finding is not that rurality is protective, nor that urban-dominant states are failing, but that state-level transplant outcome burden does not behave in a simple linear way when rurality is measured at the state level. Across 1995–2025, the higher-rurality/substantial-rural-population states had more cumulative liver transplants, fewer cumulative death removals, a lower mean death-share, and a lower death-to-transplant ratio than the urban-dominant comparators. During the period from 2020 to the present day, this trend was still observed, as the higher-rurality group once more showed a lower average death-share and a lower death-to-transplant ratio. The regression models supported this descriptive pattern: being a member of the higher-rurality group was associated with a lower death-share, the percentage of the rural population was inversely related to death-share, and the post-2020 era was independently associated with lower death-share. The critical significance of these revelations is that they substantiate that it is not always the case that a higher percentage of the rural population leads to more transplants being delayed or more people being on the death row. They instead suggest that public state-level transplant data may capture the behavior of larger transplant ecosystems, including center density, referral structure, organ supply–demand relationships, and regional care networks. Therefore, the most appropriate interpretation is ecological: these six states differed in transplant-system burden, but the analysis cannot determine whether individual rural patients within those states had better or worse access than individual urban patients.

This interpretation aligns with and also extends prior transplant-access literature. Earlier work on solid-organ transplantation demonstrated that rural residents were less likely to be waitlisted or transplanted, although post-transplant outcomes were not necessarily worse once patients entered the transplant system [13]. More recent liver-transplant literature has sharpened this concern by emphasizing that rural barriers may occur before waitlisting, during referral, or during transplant evaluation rather than only after a patient appears in national waitlist data [14,17]. Neighborhood-level deprivation and social determinants of health have also been linked to lower likelihood of waitlisting and worse outcomes during liver transplant evaluation, suggesting that geography interacts with socioeconomic position rather than functioning as a single independent exposure [15,25,26]. The Liver Outcomes and Equity Index study, for example, used national transplant data linked to American Community Survey variables and showed that neighborhood-level social determinants were associated with waitlist mortality among adult liver transplant candidates [25]. Similarly, work on area deprivation has shown that where a patient lives can predict survival and liver transplant waitlisting among patients with cirrhosis, reinforcing that geographic signals often reflect layered social and healthcare-system conditions rather than distance alone [26]. Our findings are broadly consistent with that framework, although aggregate state-level counts without per capita normalization limits the ability to fully separate geographic structure from underlying population along with disease burden differences. Instead, it shows that the scale and maturity of the state-level transplant ecosystem may shape observable public outcomes. The higher-rurality states in this analysis, especially Texas and Pennsylvania, are not small isolated rural systems; they are large, high-volume states with substantial rural populations and major transplant infrastructure.

Urban-dominant states may also appear to carry greater ecological burden for reasons that are not inherently negative and may instead reflect the consequences of being major referral hubs. Usually, large urban transplant hospitals receive patient candidates who are medically complex and medically transferred from a vast area. Such patients as are included: patients with an acute-on-chronic liver failure, patients worrying about rapidly progressive malignancy, patients having had a prior transplant, or patients with multisystem comorbidity that may not be treated as usual by other programs. These centers can also assess more socially fragile patients with a longer healthcare delay due to such problems as fragmented insurance coverage, language barriers, inadequate housing, or lack of specialty follow-up. Also, urban settings are usually characterized by areas with a high population density, which signifies that the waitlist need may still be intense even when the number of actual transplants is quite high. A state such as California may therefore perform a large number of liver transplants while simultaneously caring for a candidate pool with considerable complexity and competitive pressure. This helps explain why raw or ratio-based ecological metrics should never be interpreted as simplistic quality rankings. A higher death-share in an urban-dominant state could coexist with excellent surgical outcomes, sophisticated multidisciplinary care, and aggressive listing practices that accept higher-risk candidates. Contemporary transplant literature has repeatedly emphasized that case-mix, center behavior, and regional demand all influence observed outcomes, often in ways not fully captured by aggregate public reporting [27,28,29]. Our findings are therefore best read as signals of system strain and candidate-flow dynamics rather than judgments regarding the comparative quality of urban transplant programs.

The exclusion of Vermont, Maine, and West Virginia from inferential analysis is therefore methodologically important. These states were initially selected because they are rural-dominant by population percentage, but they did not provide stable reportable values under the project’s selected public data extraction criteria. That absence should not be dismissed as a technical inconvenience. It highlights a critical problem in geographic transplant research: the most rural states by percentage may be too low-volume, too referral-dependent, or too affected by suppression rules to appear as stable analytic units in public state-year tables. In practical terms, this means that rurality measured by state percentage and rurality experienced by patients may diverge sharply. A highly rural small state may send candidates across state lines for evaluation or transplantation, while a less rural but much larger state may contain millions of rural residents and multiple transplant centers. Public aggregate data may therefore make the most rural environments analytically faint while making high-volume mixed states appear more visible and statistically stable. This issue is especially relevant when interpreting the inverse association between rural-population percentage and death-share in our six-state sample. The association is mathematically real within the selected panel, but it should not be generalized as evidence that rural residence reduces risk. Rather, it demonstrates that ecological rurality can be confounded by state size, transplant-center capacity, referral geography, organ availability, and the denominator used to define “burden”.

The post-2020 findings deserve separate attention because they occurred during a major national allocation transition. In February 2020, Acuity Circles replaced donor service area and regional boundaries with a distance-based distribution framework intended to reduce geographic variation in access to liver transplantation [3,9]. In our analysis, the post-2020 era was associated with a substantially lower death-share and with higher annual transplant counts. These findings are directionally consistent with national reports documenting increasing liver transplant volume and with policy efforts designed to reduce geographic imbalance in allocation [1,2,3,4,9,10,11]. However, this study cannot attribute the observed post-2020 decline directly to Acuity Circles. These changes in donor utilization, center-level practice, allocation behavior, wider sharing, organ acceptance patterns, and the resolving clinical setting of liver disease were also the post-2020 period’s referring partners. On the other hand, the COVID-19 pandemic also had a significant impact on not only the performing but also the healthcare delivery sectors in ways that were state- and center-specific. A more appropriate interpretation is that death-share declined within the context of broader allocation and practice changes; the relative contribution of any single policy to this trend cannot be determined from this ecological analysis. Still, the consistency of post-2020 improvement across both groups suggests that a time-sensitive component exists and that future patient-level studies should examine whether policy-era effects differ by rural residence, distance to transplant center, and diagnosis-specific indication rather than state-level aggregates alone. The observed decline in death-share after 2020 may reflect allocation reform, increased transplant volume, changing donor utilization, center-level practice changes, or broader secular trends.

The concept of death-share is useful but must be interpreted carefully. In this study, death-share was defined as death removals divided by the sum of death removals and transplants, creating a simple ratio of adverse waitlist exit burden to the combined observed burden of death and transplantation. The main point is that it is very clear: It is possible to directly calculate it from the public state-year tables, to compare it over time, and to see it as a graph in different states easily. It is also not the case that it shows data on only death removals without considering transplant volume, which, for example, would be misleading in situations where the states compared are extremely different in size. However, death-share is not a formal OPTN endpoint, and it does not replace patient-level waitlist mortality, competing-risk transplant probability, or transplant-free survival. Additionally, it fails to consider patients who were not referred, not evaluated, not listed, withdrawn for reasons not related to death, and transplanted in other states outside their area of living. A state with a low proportion of deaths could represent the performance of effective transplantation, the presence of lower disease severity, the favorable organ availability, different listing behavior, or the incomplete capturing of upstream barriers. A state with a high death-share could reflect greater disease burden, more complex referrals, higher acuity, or a more congested transplant ecosystem. For that reason, death-share should be understood as an ecological burden signal rather than a clinical risk estimate. It is not the ability to make a causal inference that is valuable, but the generation of hypotheses and comparative descriptions.

These observations also demonstrate that ecological analyses are still essential in the field of transplant medicine. Patient-level registry studies are considered the gold standard for risk adjustment, causal inference, and the intricate examination of health equity; however, public ecological datasets stand out as useful because they allow fast data collection, clear statistical model testing, and the foundation of wider systems-level hypotheses. In the time when lawmakers and doctors are discussing the distribution of resources, center’s access, and moveable distribution, there is a considerable advantage in methods that can be reproduced without proprietary data use agreements or complex registry permissions. Ecological studies pinpoint the deviations, affirm the directional trends, or articulate the geographic clusters that warrant further investigation rooted in registry data. They can also help outside observers, including trainees and public-health investigators, participate in transplant outcomes research using accessible materials. This is particularly relevant in fields such as hepatology and transplantation, where many important datasets are restricted or expensive to access. The present study illustrates that even simple public state-year tables can reveal coherent temporal improvements, persistent between-group differences, and methodological blind spots related to data suppression in low-volume states. That does not elevate ecological data above registry science, but it does affirm a complementary role. Several health-services disciplines have long used ecological surveillance as an early-warning framework before moving toward more sophisticated patient-level investigation [30,31,32].

From a policy perspective, the current results suggest that measuring fairness in transplantation requires more than counting transplants or comparing state totals. If access is assessed only after waitlisting, then substantial barriers occurring earlier in the pathway may remain invisible. In some situations, patients might not be diagnosed with cirrhosis on time, might not have the opportunity to see a specialist hepatologist, or might be referred too late for evaluation, or even might be excluded from the workup because of the existence of one or more remediable social barriers. In addition, rural patients not only suffer from these problems but are also presented with transport costs, transport instability, employment disruption, and a shortage of caregivers that are not stated in transplant numbers only. Because of this, it would be better if forthcoming public reporting furnished statistics covering the entire process: referral volume, evaluation completion, time from referral to listing, listing to transplant interval, removals for deterioration, and post-transplant survival by rural–urban classification. Such a framework would align with broader movements in health policy that distinguish equality of final outputs from equity of opportunity across care stages [33,34]. It would also complement the evolution toward continuous distribution, where candidate prioritization is increasingly conceptualized as multidimensional rather than geographically rigid. Without upstream denominators, a state can appear successful while still failing many patients before they ever enter the official waitlist. Our study, therefore, supports a more comprehensive access dashboard rather than reliance on transplant counts alone.

Primary sclerosing cholangitis (PSC) represents one clinically relevant example of chronic liver disease in which transplantation remains an important therapeutic consideration. PSC is a progressive cholestatic liver disease associated with cirrhosis, portal hypertension, recurrent cholangitis, liver failure, and cholangiocarcinoma [18,19,20,21]. Liver transplantation remains the definitive treatment for advanced PSC, although post-transplant recurrence remains a recognized long-term challenge [18,19,20,21,22,35]. Because PSC often follows a prolonged clinical course requiring surveillance, specialty referral, multidisciplinary evaluation, and longitudinal care coordination, geographic barriers within transplant systems may be particularly relevant for affected patients. Delays in referral, long travel distances, fragmented specialty access, and dependence on regional referral networks may influence movement through the transplant pathway, especially for patients residing far from transplant centers. However, PSC was not analyzed as a diagnosis-specific cohort in the present study, and the findings should not be interpreted as PSC-specific outcome data. The current analyses instead demonstrate that state-level transplant ecosystems differ in observable waitlist outcome patterns, which may have broader implications for transplant-sensitive chronic liver diseases, including PSC. Future patient-level OPTN/SRTR studies focused specifically on PSC should evaluate rural–urban classification, transplant-center distance, MELD exception practices, inflammatory bowel disease comorbidity, and post-transplant recurrence pathways. The present study also supports a systems-level interpretation of transplantation burden rather than a psychiatric outcomes interpretation. Although psychiatric variables such as depression, anxiety, adherence, caregiver strain, and substance use were not directly measured, the transplant process itself involves substantial psychosocial and healthcare-navigation demands. Patients undergoing transplant evaluation frequently encounter repeated specialty visits, laboratory and imaging surveillance, transportation barriers, insurance coordination, caregiver dependence, and prolonged uncertainty during disease progression. These challenges may be amplified in geographically complex healthcare systems characterized by long travel distances, fragmented specialty access, and regional referral dependency. Accordingly, the psychiatric relevance of this study is systems-based and contextual rather than outcome-based, emphasizing how geography may shape the lived burden of navigating high-acuity transplant care [36,37,38].

The findings may also have implications for workforce planning and transplant infrastructure placement. By leveraging geographic spatial data, liver disease and transplant clinic geolocation revealed evidence that their spatial alignment in the United States is not always the best, thus sparking the idea that some areas continue to be the underserved ones even with the country undergoing a rise in transplant surgery [6,24,39]. Congruent with the possibility that the regions, which are characterized by high volume, essential rural places, and uphold a sort of favorable environmental metrics, have other observed networks, the active centers, or the connecting pathways, which are, in general, stronger if compared to community clinicians to the transplant programs. In contrast, communities with smaller populations or insufficiently connected technical health professionals, for instance, would likely be less equipped to deal with the situations, even though the distance is not really that far from the map viewpoint. These aforementioned initiatives must therefore serve as significant steps for the candidates who, instead, have to make long journeys on several occasions due to the existing conditions. Similar models have improved specialty access in other chronic diseases and may be particularly relevant to transplant medicine, where timing often determines candidacy [40,41]. Policymakers should also recognize that center count alone may be an incomplete marker of access if scheduling capacity, insurance acceptance, and outreach relationships remain limited. The broader lesson is that infrastructure must be functional, connected, and visible to referring clinicians. Geography matters not only because of miles traveled, but because of how systems are organized around those miles.

Several future research directions emerge directly from these findings. First, a national patient-level OPTN/SRTR analysis should test whether the inverse association observed here persists after adjustment for age, sex, race/ethnicity, MELD score, insurance type, diagnosis, center volume, and travel distance. Second, referral-denominator studies are urgently needed, because many of the most consequential barriers likely occur before listing. Third, PSC-specific cohorts should examine whether cholestatic disease pathways differ from alcohol-associated liver disease, viral hepatitis, or hepatocellular carcinoma pathways with respect to geography and timing. Fourth, linkage of registry data with census-derived deprivation indices, broadband access, transportation metrics, and clinician workforce density could clarify which components of “rurality” are most actionable. Fifth, implementation studies should evaluate whether telehealth navigation, lodging support, travel stipends, or regional PSC specialty networks improve progression from referral to transplantation. Finally, longitudinal analyses of the continuous distribution era will be important because allocation systems continue to evolve beyond the Acuity Circles framework [2,4]. The present manuscript should therefore be viewed less as a terminal answer than as a practical starting point.

### Limitations

One limitation is the relatively small number of states included in each comparison group. Although the selected states provided stable and analyzable longitudinal public data across multiple decades, grouping only six states into broad higher-rurality and urban-dominant categories may obscure important intra-group heterogeneity. Factors such as transplant-center practices, donor availability, healthcare infrastructure, funding environments, baseline population health, and disease prevalence may differ substantially even among states within the same grouping framework. Accordingly, these findings should be interpreted as exploratory ecological observations intended for hypothesis generation rather than definitive estimates of individual-level rural disadvantage. Importantly, the present analysis was not diagnosis-specific and did not isolate PSC transplant recipients within the public state-level datasets secondary to information unavailability. Accordingly, the findings should not be interpreted as direct evidence of PSC-specific disparities in transplant access or outcomes. Rather, PSC serves as a clinically important disease context because transplantation remains the definitive therapy for advanced disease and because geographic barriers affecting transplant-system navigation may plausibly influence PSC care pathways. Similarly, the ecological state-level design does not permit determination of whether individual transplant recipients resided in rural versus urban areas, nor does it capture patients who were never referred for transplant evaluation. These limitations are important because rural disadvantage may occur upstream of waitlisting and transplantation, including during referral, evaluation, transportation access, and specialty-care engagement. Several other limitations should be considered when interpreting these findings. First, the study is ecological and state-level; therefore, it cannot determine whether individual transplant recipients resided in rural or urban areas, received care at specific transplant centers, or crossed state boundaries for evaluation or transplantation. Second, the analysis used aggregate public counts and did not include ZIP-code residence, Donation Service Area, Acuity Circle-level data, transplant-center density, waitlist size, referral denominators, MELD/PELD scores, MELD exception status, diagnosis-specific etiology, race/ethnicity, insurance status, comorbidities, or socioeconomic variables. Third, death and transplantation are competing outcomes in patient-level waitlist research, but competing-risk modeling was not possible with aggregate state-year tables. Fourth, the included states contain both rural and urban populations, including major metropolitan transplant centers, so state-level rurality should not be equated with individual rural residence. These limitations mean that the findings should be interpreted as hypothesis-generating observations about transplant-system patterns rather than definitive evidence regarding individual-level rural access disparities or PSC-specific outcomes.

## 5. Conclusions

In a six-state ecological analysis using publicly available United Network for Organ Sharing (UNOS)-derived liver transplant data from 1995 to 2025, it has been found that the states with considerable rural populations (Texas, North Carolina, and Pennsylvania) had a lower death and removal burden from the waitlist than the urban-dominant comparator states (California, New Jersey, and Massachusetts), as reflected in both lower average death-share and lower death-to-transplant ratio throughout the study period and in the contemporary era after 2020. The results found in the study should not be taken as indications that the individual patients from rural areas have better chances at receiving liver transplantation; the better explanation is structural variances in the functioning of state-level transplant ecosystems, which include features such as transplant-center capacity, referral architecture, organ supply–demand balance, regional coordination among programs, and candidate case-mix. Urban-dominant states are particularly exemplified by acting as high-volume referral hubs taking in extra medically complex, socially fragile, and geographically distinct candidate pools, which may result in ecological burden metrics being elevated independently of the program quality.

The post-2020 phase was separately associated with a significant decrease in death-share and an increase in annual transplants in both groups, an occurrence that matched with the implementation of Acuity Circles allocation policy and also with the changes in donor utilization and clinical practice. Nevertheless, the ecological design of this research does not allow any attributive causal link of these improvements to a certain policy change. The exclusion of Vermont, Maine, and West Virginia from inferential modeling because of sparse or suppressed public counts further underscores a critical methodological caveat: the most rural states by population percentage may be systematically underrepresented in aggregate public reporting, meaning that rurality measured at the state level may diverge meaningfully from rurality experienced at the patient level. Although primary sclerosing cholangitis was not analyzed as a discrete sub-group in this dataset, liver transplantation remains the only definitive therapy for advanced PSC, and geographically mediated delays in referral, evaluation, listing, or transplantation may carry substantial clinical consequences for this comparatively young, medically engaged, and longitudinally complex population. Potential interventions such as telehepatology and regional referral coordination may help address upstream access barriers; however, the present study did not measure telemedicine availability or intervention uptake. These strategies should, therefore, be considered plausible future directions rather than conclusions directly supported by the present analysis [40,41].

Taken together, these results affirm the value of publicly accessible ecological analyses as a hypothesis-generating tool in transplant health-services research, while also delineating the boundaries of what such analyses can establish. Ultimately, greater evidence about the distance equity of liver transplantation will entail not only the patient-level OPTN/SRTR investigations of the nation but also the factors of rural–urban classification, travel distance to the nearest transplant center, the referral-stage proportionality measure, disease-specific cohorts (like PSC), and psychosocial determinants of access and adherence. For now, the most plausible explanation for the existing trends is that transplant systems at the state level are inequitable in ways that impact the observable waitlist outcomes, and the remaining gaps in transplant equity can be closed only by considering the entire journey from diagnosis and referral to evaluation, listing, transplantation, and long-term post-transplant care.

## Figures and Tables

**Table 1:** Study panel characteristics by state, 1995–2025.

State	Analytic Group	Rural Population %	Total Rural Population	Years Available	Total Transplants	Total Death Removals	Mean Annual Transplants	Mean Annual Death Removals	Mean Death-Share
California	Urban-dominant	5.8	1,747,542	31	25,267	7929	815.1	255.8	24.4%
Massachusetts	Urban-dominant	8.7	490,929	31	6929	2320	223.5	74.8	27.5%
New Jersey	Urban-dominant	6.2	499,036	31	1982	376	63.9	12.1	16.0%
North Carolina	Higher-rurality	33.3	3,474,661	31	5435	1075	175.3	34.7	18.0%
Pennsylvania	Higher-rurality	23.5	3,061,630	31	15,587	3626	502.8	117.0	19.1%
Texas	Higher-rurality	16.3	4,744,808	31	18,449	3941	595.1	127.1	19.1%

**Table 2:** Contemporary post-2020 outcomes by state, 2020–2025.

State	Analytic Group	Rural Population %	Years	Cumulative Transplants	Cumulative Death Removals	Mean Annual Transplants	Mean Annual Death Removals	Mean Death-Share	Death-to-Transplant Ratio
California	Urban-dominant	5.8	6	6847	976	1141.2	162.7	12.6%	0.143
Massachusetts	Urban-dominant	8.7	6	2255	364	375.8	60.7	14.9%	0.161
New Jersey	Urban-dominant	6.2	6	405	67	67.5	11.2	15.3%	0.165
North Carolina	Higher-rurality	33.3	6	1688	118	281.3	19.7	7.0%	0.070
Pennsylvania	Higher-rurality	23.5	6	3582	384	597.0	64.0	9.7%	0.107
Texas	Higher-rurality	16.3	6	5889	768	981.5	128.0	11.7%	0.130

**Table 3:** Group-level comparison across the full panel, 1995–2025.

Metric	Higher-Rurality	Urban-Dominant	Absolute Difference (Higher − Urban)	Relative Difference
Cumulative transplants, n	39,471	34,178	5293	+15.5%
Cumulative death removals, n	8642	10,625	−1983	−18.7%
Mean annual transplants	424.4	367.5	56.9	+15.5%
Mean annual death removals	92.9	114.2	−21.3	−18.7%
Mean death-share	18.7%	22.6%	−3.9 percentage points	−17.2%
Death-to-transplant ratio	0.219	0.311	−0.092	−29.6%

**Table 4:** Ecological regression models.

Model	Predictor	Coefficient	95% CI	*p*-Value
Model 1: Death-share ~ higher-rurality + post-2020	Intercept	0.2473	0.2292 to 0.2654	<0.001
	Higher-rurality group	−0.0389	−0.0604 to −0.0175	<0.001
	Post-2020	−0.1091	−0.1299 to −0.0882	<0.001
	Model R^2^	0.290	—	—
Model 2: Death-share ~ rural % + post-2020	Intercept	0.2536	0.2311 to 0.2761	<0.001
	Rural population %	−0.001649	−0.002837 to −0.000461	0.007
	Post-2020	−0.1091	−0.1298 to −0.0883	<0.001
	Model R^2^	0.276	—	—
Model 3: Annual transplants ~ higher-rurality + post-2020	Intercept	324.8	258.1 to 391.4	<0.001
	Higher-rurality group	56.9	−25.0 to 138.8	0.173
	Post-2020	220.8	86.9 to 354.8	0.001
	Model R^2^	0.095	—	—

## Data Availability

The original data presented in the study are openly available in UNOS at https://hrsa.unos.org/data/view-data-reports/state-data/?type=state&selectAreaValue=CA# (accessed on 17 April 2026) and https://hrsa.unos.org/data/view-data-reports/center-data/?type=center&selectAreaValue=AK# (accessed on 17 April 2026).
